# Outcomes of Patients with Gastrointestinal Stromal Tumors in the Past Decade

**DOI:** 10.3390/medsci11030054

**Published:** 2023-08-28

**Authors:** Ayrton Bangolo, Pierre Fwelo, Tha’er Al-Qatish, John Bukasa-Kakamba, Tiffany Lee, Akira G. Cayago, Sarah Potiguara, Vignesh K. Nagesh, Jessica Kawall, Rashid Ahmed, Muhammad Asjad Abbas, Narissa Nursjamsi, Stacy H. Lee, Shagi Meti, Georgemar V. Arana, Chrishanti A. Joseph, Abdifitah Mohamed, Arthur Alencar, Huzaifa G. Hassan, Pramanu Aryal, Aleena Javed, Maksim Kalinin, Gbenga Lawal, Ibtihal Y. Khalaf, Midhun Mathew, Praveena Karamthoti, Bhavna Gupta, Simcha Weissman

**Affiliations:** 1 Department of Medicine, Hackensack Meridian Health/Palisades Medical Center, North Bergen, NJ 07047, USA; thaerq@gmail.com (T.A.-Q.); sarahp@gmail.com (S.P.); georgemarara@gmail.com (G.V.A.); chrishantijo@gmail.com (C.A.J.); maksika@gmail.com (M.K.); gbengala@gmail.com (G.L.); ibtihalkal@gmail.com (I.Y.K.);; 2 Department of Epidemiology, Human Genetics and Environmental Sciences, UTHealth School of Public Health, Houston, TX 77204, USA; 3 Division of Endocrinology, Department of Medicine, Kinshasa University Clinics, Kinshasa 7948, Democratic Republic of the Congo; johnbukasa73@gmail.com; 4 Department of Medicine, University of Washington, Seattle, WA 98195, USA; 5 Division of Hematology and Oncology, Department of Medicine, Hackensack Meridian Health/Palisades Medical Center, North Bergen, NJ 07047, USA

**Keywords:** GISTs, GI neurotransmitter, pacemaker, SEER database, clinical characteristics, mortality

## Abstract

Background: Gastrointestinal stromal tumors (GISTs) are rare mesenchymal neoplasms of the gastrointestinal tract (GIT) that represent approximately 1 to 2 percent of primary gastrointestinal (GI) cancers. Owing to their rarity, very little is known about their overall epidemiology, and the prognostic factors of their pathology. The current study aimed to evaluate the independent determinants of mortality in patients diagnosed with GISTs over the past decade. Methods: Our study comprised 2374 patients diagnosed with GISTs from 2000 to 2017 from the Surveillance, Epidemiology, and End Results (SEER) database. We analyzed the baseline characteristics, and overall mortality (OM), as well as the cancer-specific mortality (CSM) of GISTs. Variables with a *p* value < 0.01 in the univariate Cox regression were incorporated into the multivariate Cox model, to determine the independent prognostic factors. Results: Multivariate Cox proportional hazard regression analyses of factors affecting the all-cause mortality and GIST-related mortality among US patients between 2010 and 2017 revealed a higher overall mortality in non-Hispanic Black patients (HR = 1.516, 95% CI 1.172–1.961, *p* = 0.002), patients aged 80+ (HR = 9.783, 95% CI 4.185–22.868, *p* = 0), followed by those aged 60–79 (HR = 3.408, 95% CI 1.488–7.807, *p* = 0.004); male patients (HR = 1.795, 95% CI 1.461–2.206, *p* < 0.001); patients with advanced disease with distant metastasis (HR = 3.865, 95% CI 2.977–5.019, *p* < 0.001), followed by cases with regional involvement via both direct extension and lymph node involvement (HR = 3.853, 95% CI 1.551–9.57, *p* = 0.004); and widowed patients (HR = 1.975, 95% CI 1.494–2.61, *p* < 0.001), followed by single patients (HR = 1.53, 95% CI 1.154–2.028, *p* = 0.003). The highest CSM was observed in the same groups, except widowed patients and patients aged 60–79. The highest CSM was also observed among patients that underwent chemotherapy (HR = 1.687, 95% CI 1.19–2.392, *p* = 0.003). Conclusion: In this updated study on the outcomes of patients with GISTs, we found that non-Hispanic Black patients, male patients, and patients older than 60 years have a higher mortality with GISTs. Furthermore, patients who have received chemotherapy have a higher GIST-specific mortality, and married patients have a lower mortality. However, we do not know to what extent these independent prognostic factors interact with each other to influence mortality. This study paves the way for future studies addressing these interactions. The results of this study may help treating clinicians to identify patient populations associated with a dismal prognosis, as those may require closer follow-up and more intensive therapy; furthermore, with married patients having a better survival rate, we hope to encourage clinicians to involve family members of the affected patients early in the disease course, as the social support might impact the prognosis.

## 1. Introduction

A gastrointestinal stromal tumor (GIST) represents a distinct entity from other mesenchymal tumors of the GIT, with an immunohistochemistry profile that differs from that of leiomyomas and leiomyosarcomas arising from other sites, such as the uterus or soft tissues [1,2,3]. GISTs are thought to originate from the interstitial cells of Cajal (ICCs), which regulate peristalsis by forming the interface between the autonomic innervation of the bowel wall and the smooth muscle itself [4]. GISTs can occur anywhere in the GIT, from the esophagus to the anus [5]. 

Most cases of GISTs are sporadic, arising from de novo mutation. However, approximately 5 percent of patients with GISTs have one of several genetic syndromes associated with the development of these tumors, including primary familial GIST syndrome, neurofibromatosis type 1 (NF1), Carney–Stratakis syndrome, and Carney triad [6]. A GIST, in most cases, carries a mutation in the KIT or platelet-derived growth factor receptor-alpha (PDGFRA) genes. However, there is a subset of GISTs called “wildtype”, which have no detectable mutations on the KIT or PDGFRA genes [6]. A GIST’s initial symptoms will vary based on the involved primary site; GI bleeding may be the presenting symptom for upper GISTs, such as those affecting the stomach, small intestine, or esophagus [5]. Dysphagia and jaundice can also be observed with upper GISTs. Colorectal GISTs may present with constipation or bowel obstruction. Some male patients can experience urinary hesitancy, as a result of the tumor pushing on the prostate gland [5]. 

A GIST is often diagnosed incidentally, so the true incidence of this disease may be difficult to determine accurately [6,7]. The imaging of choice to establish a GIST diagnosis is computed tomography (CT) of the abdomen and pelvis, with oral and intravenous (IV) contrast to help define bowel margins, and assess the extent of the primary mass, including local invasion into adjacent structures [8]. Magnetic resonance imaging (MRI) can be used in patients that can tolerate CT, or are allergic to CT contrast [8]. Although CT is better than MRI at visualizing the small intestine thickness and bowel perforation, MRI is preferred for primary rectal GISTs [8]. Upper endoscopy with endoscopic ultrasound (EUS) or colonoscopy may be used to further assist the diagnosis of GISTs, depending on the location. EUS can be used to diagnose upper GISTs (esophagus, stomach, and small intestine), whereas colonoscopy will aid in diagnosing lower GISTs (colon, rectum, and anus). Surgical resection remains the mainstay of the treatment of GISTs [9]. 

Only a few studies have addressed the overall epidemiology of GISTs [5,9,10,11]. However, there is still a paucity of conclusive data, and a lack of adequately powered studies properly defining the epidemiology characteristics, survival outcomes, and prognostic factors of patients with GISTs over the past decade. This is especially important in the era of the emergence of adjuvant and neoadjuvant therapies, such as imatinib [12,13]. 

Using a nationally representative and well-updated database, we evaluated the independent prognostic factors among patients with GISTs, to fill in the gap in the literature. Using this study as a path, larger prospective studies can be carried out, focusing on the independent prognostic factors of GISTs, and the interaction between them. Furthermore, we aimed to establish patient populations that are predisposed to have a poorer prognosis. These patients may need closer follow up and more aggressive therapy, especially in this era of newer therapeutics.

## 2. Materials and Methods

### 2.1. Study Design

A population-based retrospective cohort study of patients with a GIST diagnosis was conducted using the SEER Research Plus data, 18 registries, and the November 2020 submission database (http://www.seer.cancer.gov) accessed on 29 June 2022. The SEER Program is one of the largest and most authoritative sources of cancer-related datasets in the United States, and is sponsored by the United States National Cancer Institute (US NCI). The SEER 18 database collects cancer incidence, patients’ clinicopathological features, and survival data from 18 population-based cancer registries, and covers nearly 28% of the US population [14].

### 2.2. Data Selection

#### 2.2.1. Inclusion Criteria

Histological codes matching the diagnosis of GISTs, and topographical codes matching different GI locations were used to retrieve data from the SEER database [14]. 

#### 2.2.2. Exclusion Criteria

We excluded patients with an unknown age at diagnosis, race, or stage of GIST development. Therefore, 12,371 were excluded from our study. 

### 2.3. Study Variables

#### 2.3.1. Main Exposure

All the variables used in this study were the main exposures. 

#### 2.3.2. Outcomes

The OM (overall mortality) is defined as any cause of death by the end of this study period.

CSM refers to patients who had died from GIST complications by the end of this study. 

#### 2.3.3. Sociodemographic and Tumor Characteristics

Variables such as the age at diagnosis, gender, race (White, Black, and others), origin (non-Hispanic and Hispanic), primary site of the tumor, stage at diagnosis (localized, regional, and distant), geographic residential area, yearly income, marital status, year of diagnosis, treatment via surgery and/or radiation, as well as chemotherapy, were extracted. 

### 2.4. Statistical Analysis

The Cox proportional hazard regression model is based on the assumption that hazard rates are proportional over time. Variables with a value <0.1 in the univariate Cox regression model were incorporated into the multivariate Cox proportional analysis, to determine the independent prognostic factors associated with OM and CSM, with a hazard ratio (HR) > 1 representing adverse prognostic factors. All tests were two-sided, with a confidence interval set as 95%, and *p* value < 0.01 deemed statistically significant. All statistical tests were performed using the software STATA 16.1.

## 3. Results

We identified 2374 patients with a GIST diagnosis in our cohort, and the baseline characteristics of patients can be found in Table 1. Patients of male and female gender were almost equally affected (50.34% vs. 49.66%). Most patients were diagnosed between the ages of 60 and 79 (53.37%). Non-Hispanic Whites represented the majority of the cohort (56.19%), and the stomach was the most affected primary location (65.71%). Most tumors were diagnosed at the localized stage (80.45%). People living in counties in metropolitan areas of 1 million people (58.55%), people with an annual income of USD 75,000+ (31.89%), and married patients (57.96%) were more likely to be diagnosed than their counterparts. A total of 853 (35.93%) underwent chemotherapy, and only 4 (0.17%) underwent radiation. 

The crude analysis of factors associated with the all-cause mortality and GIST-related mortality among US patients between 2010 and 2017 is demonstrated in Table 2. Male patients (HR = 1.532, 95% CI 1.27–1.847, *p* = 0), patients aged 80+ (HR = 10.778, 95% CI 4.741–24.502, *p* = 0), followed by those aged 60–79 (HR = 3.723, 95% CI 1.657–8.367, *p* = 0.001); non-Hispanic Black patients (HR = 1.3, 95% CI 1.041–1.623, *p* = 0.02); GIST cases with distant metastases (HR = 3.765, 95% CI 3.018–4.695, *p* < 0.001); nonmetropolitan counties adjacent to a metropolitan area (HR = 1.535, 95% CI 1.099–2.146, *p* = 0.012); widowed patients (HR = 2.496, 95% CI 1.953–3.191, *p* < 0.001), and those who have undergone chemotherapy (HR = 1.29, 95% CI 1.071–1.554, *p* = 0.007) have the highest overall mortality. The highest cancer specific mortality was observed in the same groups. 

The multivariate Cox proportional hazard regression analyses of factors affecting thr all-cause mortality and GIST-related mortality among US patients between 2010 and 2017 are demonstrated in Table 3. A higher overall mortality was observed in non-Hispanic Blacks (HR = 1.516, 95% CI 1.172–1.961, *p* = 0.002), patients aged 80+ (HR = 9.783, 95% CI 4.185–22.868, *p* = 0), followed by those aged 60–79 (HR = 3.408, 95% CI 1.488–7.807, *p* = 0.004); male patients (HR = 1.795, 95% CI 1.461–2.206, *p* < 0.001); cases of advanced disease with distant metastasis (HR = 3.865, 95% CI 2.977–5.019, *p* < 0.001), followed by regional involvement via both direct extension and lymph node involvement (HR = 3.853, 95% CI 1.551–9.57, *p* = 0.004); and widowed patients (HR = 1.975, 95% CI 1.494–2.61, *p* = 0), followed by single patients (HR = 1.53, 95% CI 1.154–2.028, *p* = 0.003). The highest CSM was observed in the same groups, except widowed patients and patients aged 60–79. The highest CSM was also observed among patients who underwent chemotherapy (HR = 1.687, 95% CI 1.19–2.392, *p* = 0.003). The cancer site, yearly income, and treatment with radiation therapy did not show a significant association with the all-cause mortality or cancer-specific mortality.

## 4. Discussion 

Based on this nationally representative large study, we found that non-Hispanic Whites are more likely to be affected by GISTs. The stomach is the most commonly affected site, and most patients are diagnosed at a localized stage of the disease. The majority of patients did not undergo chemotherapy. Non-Hispanic Blacks, older patients, male patients, cases of an advanced disease state, and single/widowed patients had a higher mortality. Furthermore, patients who underwent chemotherapy seemed to have a higher CSM. 

Most patients in our cohort were diagnosed between the ages of 60 and 79 years, which overlaps with the findings in the literature, where a median age of diagnosis was found to be between 65 and 69 years [9,15,16]. The male-to-female ratio in our cohort was found to be almost 1:1, which is in accordance with the current literature [9]. However, non-Hispanic White populations were the most affected in the past decade alone, which contrasts with the current literature, where the most affected ethnicity is African American [5,17]. A study by Ulanja et al. on the racial disparity on the incidence of GISTs, using the SEER database, over a period from 2002 to 2015 demonstrated that GISTs were more common among African Americans [17]. We unveiled that, over the past two decades, non-Hispanic Whites showed more cases than non-Hispanic Blacks; however, while considering the last decade alone, the opposite has held true. 

Our cohort found that GISTs were most likely diagnosed in metropolitan areas with more than 1 million people, and among patients earning USD 75,000+. GISTs can present with nonspecific symptoms, such as occult gastrointestinal (GI) bleeding, asymptomatic presentation, abdominal discomfort, an acute abdomen, or an asymptomatic abdominal mass [18,19,20,21,22]. Thus, GISTs can be detected during an endoscopic study; elective surgical procedures (e.g., sleeve gastrectomy for patients with obesity); or on imaging performed for another purpose [23]. Therefore, multiple visits to the physician, or access to advanced surgical or endoscopic techniques, may be required before the diagnosis can be made. People living in metropolitan areas are more likely to have access to advanced surgical and endoscopic techniques. Furthermore, imaging such as computed tomography (CT), magnetic resonance imaging (MRI), or positron emission tomography (PET) scanning using fluorodeoxyglucose (FDG–PET), in combination with CT (PET–CT), can be used to evaluate the primary tumor [24]. These imaging modalities can be quite expensive, and will be affordable to patients with a higher yearly income. 

Our study did not find any mortality differences among the different primary sites affected with GISTs. Contrastingly, a study by Miettinen et al. revealed that the gastric location had more favorable survival outcomes compared with those arising from other sites in the GI tract [25]. Equivalent results were found in studies by Khan et al. and Kukar et al., where a non-gastric/non-small-intestinal location (the esophagus and ascending and sigmoid colon) was associated with worse overall survival [15,26]. Older patients had a higher mortality in our cohort, for both OM and CSM. These findings mirror those found in the literature, where patients older than 60 years of age had a worse survival outcome compared to their younger counterparts, likely pertaining to their weak immune system [15,27]. 

Non-Hispanic Black patients were found to have a higher OM and CSM compared to other races. This finding contrasts with the findings of the studies conducted by Ulanja et al., and the analysis performed by Cheung et al. after 2000 [17,28]. However, Cheung et al. found a higher mortality rate in African American patients compared to other races before the year 2000, and this was thought to be due to the lower rates of surgical excision of the primary tumor among African Americans [28]. Male patients were found to have a worse outcome compared to their female counterparts, which is consistent with current findings in the literature [29]. 

Marital status has been portrayed as an independent prognostic factor in several series around the globe, with married patients having a favorable outcome [30,31,32,33,34,35,36,37,38,39]. An explanation for this observation could be a better level of social assistance among married patients. The current study indeed found that single patients had a higher CSM and overall mortality. Furthermore, widowed patients were found to have a higher OM. Using these findings, we hope to encourage clinicians (oncologists and primary care physicians) to involve family members of non-married patients early during the disease course, as the social support may help improve the survival of patients with GISTs.

Patients who underwent chemotherapy were found to have a higher CSM, in our cohort. This contrasts with the literature, where the use of chemotherapies, either as an adjuvant or neoadjuvant treatment, such as tyrosine kinase inhibitors (imatinib), have been associated with better overall survival [40]. This could be explained by the toxic effects of chemotherapy. Furthermore, imatinib is used as a neoadjuvant therapy before surgery for large tumors and advanced stages of GISTs, which is associated with a higher mortality [41]. Thus, treating physicians should weigh the true benefit of starting such patients on chemotherapy against the adverse effects of the therapy. 

Certain limitations must be considered in the interpretation of the results of this study. Information on patients who underwent surgery was not used in our cohort, as the information available was reported as either “yes” or “no/unknown”. The information available for chemotherapy did not specify if this treatment was given as neoadjuvant or adjuvant. Furthermore, the SEER database, the largest cancer database in the USA that is publicly available, does not provide information on comorbidities. 

## 5. Conclusions

To summarize the findings of this original study, as one would expect, older patients, and those with advanced disease, had a worse prognosis. Furthermore, we found that non-Hispanic Blacks, male patients, and those who have received chemotherapy have a higher mortality with GISTs, whereas married patients have a lower mortality. These data can assist treating oncologists and/or primary care physicians in identifying specific patient populations that may need a closer follow up, and a more aggressive therapy. In an effort to improve the survival of non-married patients, we hope to encourage clinicians to be on the lookout for a robust social support for patients with GISTs, as this could play a determinant role in lowering mortality. We propose that elderly Black male patients with a GIST diagnosis should be closely followed up, compared with other patient groups. This study paves the way for future studies addressing the interaction between the independent prognosis factors found in this cohort.

## Figures and Tables

**Table 1:** Demographic and clinicopathologic characteristics of US patients with a GIST diagnosis between 2000 and 2017.

Characteristics		
	*N=*	%
Total	2374	100.00
Gender		
Female	1179	49.66
Male	1195	50.34
Age at diagnosis, y.o.		
0–39	108	4.55
40–59	736	31.00
60–79	1267	53.37
80+	263	11.08
Race		
Non-Hispanic White	1334	56.19
Non-Hispanic Black	466	19.63
Hispanic	279	11.75
Other	295	12.43
Cancer site		
Colon	40	1.68
Esophagus	10	0.42
Stomach	1560	65.71
Rectum	39	1.64
Small intestine	706	29.74
Other	19	0.80
Tumor stage		
Localized	1910	80.45
Regional via direct extension only	204	8.59
Regional lymph nodes involved only	22	0.93
Regional via both direct extension and lymph node involvement	13	0.55
Distant	224	9.44
Living area		
Counties in metropolitan areas of 1 million persons	1390	58.55
Counties in metropolitan areas of 250,000 to 1 million persons	592	24.94
Counties in metropolitan areas of 250,000 persons	134	5.64
Nonmetropolitan counties adjacent to a metropolitan area	163	6.87
Nonmetropolitan counties not adjacent to a metropolitan area	95	4.00
Income per year		
USD < 35,000	46	1.94
USD 35,000–44,999	166	6.99
USD 45,000–54,999	346	14.57
USD 55,000–64,999	563	23.72
USD 65,000–74,999	496	20.89
USD 75,000+	757	31.89
Marital status		
Married	1376	57.96
Single	397	16.72
Divorced/separated	237	9.98
Widowed	258	10.87
Unknown	106	4.47
Radiation		
No	2370	99.83
Yes	4	0.17
Chemotherapy		
No	1521	64.07
Yes	853	35.93
Year of diagnosis		
2010	186	7.83
2011	209	8.80
2012	278	11.71
2013	267	11.25
2014	307	12.93
2015	333	14.03
2016	386	16.26
2017	408	17.19

**Table 2:** Crude analysis of factors associated with the all-cause mortality and GIST-related mortality among US patients between 2000 and 2017.

Characteristics	Overall Mortality. Crude Proportional Hazard Ratio (95% Confidence Interval)	GIST Mortality. Crude Proportional Hazard Ratio (95% Confidence Interval)
Gender		
Female	1 (reference)	1 (reference)
Male	1.53 (1.27–1.85) ***	1.46 (1.09–1.95) **
Age at diagnosis, y.o.		
0–39	1 (reference)	1 (reference)
40–59	1.92 (0.84–4.39)	1.18 (0.51–2.75)
60–79	3.72 (1.66–8.37) ***	1.45 (0.64–3.32)
80+	10.78 (4.74–24.50) ***	3.08 (1.29–7.37) **
Race		
Non-Hispanic White	1 (reference)	1 (reference)
Non-Hispanic Black	1.30 (1.04–1.62) **	1.77 (1.27–2.47) ***
Hispanic	0.86 (0.62–1.20)	1.04 (0.63–1.73)
Other	0.78 (0.57–1.08)	1.03 (0.64–1.66)
Cancer site		
Colon	1 (reference)	1 (reference)
Esophagus	1.55 (0.41–5.86)	1.02 (0.11–9.15)
Stomach	0.92 (0.45–1.85)	0.65 (0.24–1.76)
Rectum	0.80 (0.29–2.20)	0.94 (0.23–3.74)
Small intestine	1.08 (0.53–2.21)	1.09 (0.40–2.97)
Other	0.76 (0.20–2.86)	1.00 (0.18–5.47)
Tumor stage		
Localized	1 (reference)	1 (reference)
Regional via direct extension only	1.41 (1.03–1.93) **	2.54 (1.62–3.99) ***
Regional lymph nodes involved only	0.64 (0.16–2.56)	0
Regional via both direct extension and lymph node involvement	3.03 (1.25–7.34) **	6.19 (1.96–19.57) ***
Distant	3.77 (3.02–4.70) ***	8.55 (6.26–11.68) ***
Living area		
Counties in metropolitan areas of 1 million persons	1 (reference)	1 (reference)
Counties in metropolitan areas of 250,000 to 1 million persons	1.12 (0.90–1.40)	1.01 (0.70–1.44)
Counties in metropolitan areas of 250,000 persons	1.24 (0.86–1.79)	1.66 (1.01–2.75) **
Nonmetropolitan counties adjacent to a metropolitan area	1.54 (1.10–2.15) **	1.68 (1.01–2.77) **
Nonmetropolitan counties not adjacent to a metropolitan area	1.34 (0.88–2.04)	1.22 (0.62–2.42)
Income per year		
USD < 35,000	1 (reference)	1 (reference)
USD 35,000–44,999	1.82 (0.90–3.69)	2.56 (0.77–8.47)
USD 45,000–54,999	1.21 (0.61–2.42)	1.27 (0.39–4.19)
USD 55,000–64,999	1.20 (0.61–2.37)	1.77 (0.56–5.64)
USD 65,000–74,999	0.90 (0.45–1.81)	0.95 (0.29–3.15)
USD 75,000+	0.92 (0.46–1.80)	1.03 (0.32–3.33)
Marital status		
Married	1 (reference)	1 (reference)
Single	1.18 (0.91–1.54)	1.44 (0.99–2.10)
Divorced/separated	1.12 (0.81–1.55)	1.11 (0.67–1.83)
Widowed	2.50 (1.95–3.19) ***	1.64 (1.06–2.55) **
Radiation		
No	1 (reference)	1 (reference)
Yes	4.00 (1.00–16.07)	4.75 (0.67–33.94)
Chemotherapy		
No	1 (reference)	1 (reference)
Yes	1.29 (1.07–1.55) ***	2.83 (2.11–3.80) ***

*** *p* < 0.01, ** *p* < 0.05.

**Table 3:** Multivariate Cox proportional hazard regression analyses of factors affecting the all-cause mortality and GIST-related mortality among US patients between 2000 and 2017.

Characteristics	Overall Mortality. Adjusted Proportional Hazard Ratio (95% Confidence Interval)	GIST Mortality. Adjusted Proportional Hazard Ratio (95% Confidence Interval)
Gender		
Female	1 (reference)	1 (reference)
Male	1.80 (1.46–2.21) ***	1.53 (1.11–2.11) **
Age at diagnosis, y.o.		
0–39	1 (reference)	1 (reference)
40–59	1.66 (0.71–3.85)	1.01 (0.42–2.43)
60–79	3.41 (1.49–7.81) ***	1.41 (0.59–3.37)
80+	9.78 (4.19–22.87) ***	3.89 (1.54–9.84) ***
Race		
Non-Hispanic White	1 (reference)	1 (reference)
Non-Hispanic Black	1.52 (1.17–1.96) ***	2.17 (1.44–3.27) ***
Hispanic	1.08 (0.77–1.53)	1.07 (0.63–1.81)
Other	1.05 (0.74–1.48)	1.49 (0.89–2.50)
Cancer site		
Colon	1 (reference)	1 (reference)
Esophagus	2.67 (0.68–10.58)	3.37 (0.34–33.60)
Stomach	1.08 (0.50–2.30)	0.89 (0.28–2.83)
Rectum	1.17 (0.40–3.41)	1.67 (0.36–7.71)
Small intestine	1.24 (0.57–2.69)	1.37 (0.42–4.47)
Other	0.86 (0.22–3.36)	0.92 (0.15–5.76)
Tumor stage		
Localized	1 (reference)	1 (reference)
Regional via direct extension only	1.5 (1.08–2.09) **	2.17 (1.33–3.52) ***
Regional lymph nodes involved only	0.83 (0.20–3.39)	0
Regional via both direct extension and lymph node involvement	3.85 (1.55–9.57) ***	5.55 (1.70–18.17) ***
Distant	3.865 (2.977–5.019) ***	6.586 (4.534–9.567) ***
Living area		
Counties in metropolitan areas of 1 million persons	1 (reference)	1 (reference)
Counties in metropolitan areas of 250,000 to 1 million persons	1.05 (0.82–1.34)	0.98 (0.66–1.46)
Counties in metropolitan areas of 250,000 persons	0.98 (0.65–1.48)	1.28 (0.70–2.31)
Nonmetropolitan counties adjacent to a metropolitan area	1.09 (0.72–1.65)	1.36 (0.73–2.54)
Nonmetropolitan counties not adjacent to a metropolitan area	1.15 (0.70–1.91)	1.871 (0.83–4.24)
Income per year		
USD < 35,000	1 (reference)	1 (reference)
USD 35,000–44,999	1.63 (0.75–3.56)	3.06 (0.70–13.46)
USD 45,000–54,999	1.26 (0.60–2.77)	2.38 (0.53–10.62)
USD 55,000–64,999	1.28 (0.58–2.81)	3.23 (0.73–14.36)
USD 65,000–74,999	0.95 (0.42–2.16)	1.72 (0.37–8.09)
USD 75,000+	1.04 (0.47–2.35)	2.35 (0.51–10.87)
Marital status		
Married	1 (reference)	1 (reference)
Single	1.53 (1.15–2.03) ***	1.65 (1.10–2.48) **
Divorced/separated	1.41 (1.00–1.97)	1.34 (0.79–2.29)
Widowed	1.98 (1.49–2.61) ***	1.49 (0.91–2.46)
Radiation		
No	1 (reference)	1 (reference)
Yes	1.47 (0.33–6.50)	0.75 (0.09–6.27)
Chemotherapy		
No	1 (reference)	1 (reference)
Yes	0.99(0.79–1.23)	1.69 (1.19–2.39) ***

*** *p* < 0.01, ** *p* < 0.05.

## Data Availability

The data used and/or analyzed in this study are available in the Surveillance, Epidemiology, and End Results (SEER) Database of the National Cancer Institute (http://seer.cancer.gov, accessed on 29 June 2022).
